# Novel receptor, mutation, vaccine, and establishment of coping mode for SARS-CoV-2: current status and future

**DOI:** 10.3389/fmicb.2023.1232453

**Published:** 2023-08-14

**Authors:** Zhaomu Zeng, Xiuchao Geng, Xichao Wen, Yueyue Chen, Yixi Zhu, Zishu Dong, Liangchao Hao, Tingting Wang, Jifeng Yang, Ruobing Zhang, Kebin Zheng, Zhiwei Sun, Yuhao Zhang

**Affiliations:** ^1^Department of Neurosurgery, Jiangxi Provincial People’s Hospital, The First Affiliated Hospital of Nanchang Medical College, Nanchang, China; ^2^Department of Neurosurgery, Xiangya Hospital Jiangxi Hospital of Central South University, National Regional Medical Center for Nervous System Diseases, Nanchang, China; ^3^Department of Neurosurgery, Affiliated Hospital of Hebei University, Baoding, China; ^4^Department of Nursing, School of Medicine, Taizhou University, Taizhou, China; ^5^Department of Toxicology and Sanitary Chemistry, School of Public Health, Capital Medical University, Beijing, China; ^6^Department of Pharmacy, The Second Affiliated Hospital of Nanchang University, Nanchang, China; ^7^Department of Zoology, Advanced Research Institute, Jiangxi University of Chinese Medicine, Nanchang, China; ^8^Department of Plastic Surgery, Shaoxing People’s Hospital, Shaoxing, China; ^9^Cancer Center, Department of Neurosurgery, Zhejiang Provincial People’s Hospital (Affiliated People’s Hospital), Hangzhou Medical College, Hangzhou, China

**Keywords:** Omicron variant, entry receptors, attachment factors, COVID-19 vaccines, heterologous booster immunization, small molecule antiviral drugs

## Abstract

Since the outbreak of severe acute respiratory syndrome coronavirus 2 (SARS-CoV-2) and its resultant pneumonia in December 2019, the cumulative number of infected people worldwide has exceeded 670 million, with over 6.8 million deaths. Despite the marketing of multiple series of vaccines and the implementation of strict prevention and control measures in many countries, the spread and prevalence of SARS-CoV-2 have not been completely and effectively controlled. The latest research shows that in addition to angiotensin converting enzyme II (ACE2), dozens of protein molecules, including AXL, can act as host receptors for SARS-CoV-2 infecting human cells, and virus mutation and immune evasion never seem to stop. To sum up, this review summarizes and organizes the latest relevant literature, comprehensively reviews the genome characteristics of SARS-CoV-2 as well as receptor-based pathogenesis (including ACE2 and other new receptors), mutation and immune evasion, vaccine development and other aspects, and proposes a series of prevention and treatment opinions. It is expected to provide a theoretical basis for an in-depth understanding of the pathogenic mechanism of SARS-CoV-2 along with a research basis and new ideas for the diagnosis and classification, of COVID-19-related disease and for drug and vaccine research and development.

## 1. Introduction

Since the outbreak of coronavirus disease 2019 (COVID-19) at the end of 2019, it has rapidly spread to almost all countries and regions in the world and become a major global public health threat (Thakur and Ratho, 2022). SARS-CoV-2 has been evolving and mutating continuously, resulting in more than 1,000 variant strains worldwide (Cosar et al., 2022). Because the mutation of the virus is a decisive factor in future trends of the COVID-19 pandemic, the emergence of mutant strains has aroused widespread concern in society. To strengthen the monitoring and tracking of SARS-CoV-2 variants, the World Health Organization (WHO) classified them into variants of concern (VOCs) and variants of interest (VOIs) according to their transmissibility and pathogenicity. Among them, VOCs are the variant strains with the greatest impact on the global pandemic, including Alpha, Beta, Gamma, Delta, and Omicron (Chen K. et al., 2022; Salehi-Vaziri et al., 2022; Table 1). To date, although the pathogenicity of Omicron variants seems to be gradually decreasing, their virus transmission and susceptibility are gradually increasing. Especially for children, elderly individuals or patients with underlying diseases, SARS-CoV-2 infection is still a major health threat (Mistry et al., 2021; Pellegrino et al., 2022). According to the real-time statistics of Johns Hopkins University in the United States, the number of confirmed cases of COVID-19 in the world has exceeded 670 million, and unfortunately, over 6.8 million people have died (Johns Hopkins University, 2022). It is very important to understand the virological characteristics of SARS-CoV-2 variants for the prevention and control of the COVID-19 pandemic.

**TABLE 1:** Basic overview and main characteristics of five VOCs.

WHO lable	Pango lineage	First detected in the country/Detection date	Status	Spike protein substitutions	Attributes
Alpha	B.1.1.7	United Kingdom/ September-2020	VOC:18-December-2020	H69del/V70del/Y144del/N501Y/A570D/ D614G/P681H/T716I/S982A/D1118H	Attack the immune system, with enhanced transmission and increased virulence
Beta	B.1.351	South Africa/ May-2020	VOC:18-December-2020	L18F/D80A/D215G/L242del/A243del/ L244de/R246I/K417N/E484K/N501Y/ D614G/A701V	It is highly contagious and can avoid vaccines and immune cell tracking, but the transmission power is weak
Gamma	P.1	Brazil/ November-2020	VOC:11-January-2021	L18F/T20N/P26S/D138Y/R190S/K417T/ E484K/N501Y/D614G/H655Y/T1027I/ V1176F	Damage immunity and enhance transmission
Delta	B.1.617.2	India/ October-2020	VOI: 4-April-2021 VOC:11-May-2021	T19R/G142D/E156del/F157del/R158G/ L452R/T478K/D614G/P681R/D950N	The transmission speed is fast, the viral load is high, and the infection ability is extremely strong
Omicron	B.1.1.529	India/ October-2020	VUM:24-November-2021 VOC:26-November-2021	A67V/H69del/V70del/T95I/G142D/ V143del/Y144del/Y145del/N211del/ L212I/ins214EPE/G339D/S371L/S373P/ S375F/K417N/N440K/G446S/S477N/ T478K/E484A/Q493R/G496S/Q498R/ N501Y/Y505H/T547K/D614G/H655Y/ N679K/P681H/N764K/D796Y/N856K/ Q954H/N969K/L981F	Strong transmissibility and immune evasion ability, less virulence

Governments and scientists have made many efforts to prevent, control and treat COVID-19 and have found some suitable control methods including mRNA vaccines (Naseri et al., 2022). Previous studies have shown that angiotensin-converting enzyme II (ACE2) is the main membrane receptor for SARS-CoV-2 entry into host cells (Pandey et al., 2021). However, with further follow-up study, dozens of newly discovered protein receptors have been found to mediate or assist SARS-CoV-2 in invading human cells (Ge et al., 2021; Wang J. et al., 2021), indicating SARS-CoV-2 may have many complex pathogenic mechanisms and providing new research directions for finding or developing effective antiviral drugs. In addition, with the continuous observed mutation and immune evasion of viruses, the research and development of related vaccines is also facing great challenges (Jafari et al., 2022; Pellegrino et al., 2022).

In this paper, we comprehensively reviewed the genomic characteristics, pathogenesis and latest research on SARS-CoV-2 attacking human cells through multiple receptors other than ACE2. This article comprehensively introduces the genomic characteristics of SARS-CoV-2, as well as the latest research progress on human cell infection based on ACE2 and various other receptor pathways. In addition, we summarize the virus mutations (especially the Omicron variant) and vaccine development and finally propose some new perspectives on responding to the SARS-CoV-2 pandemic. We hope this review can provide a literature basis for a better understanding of the molecular characteristics and pathogenic mechanism of SARS-CoV-2 and lay a theoretical foundation for precise diagnosis, specific drug development, and vaccine development of related diseases caused by SARS-CoV-2 infection.

## 2. Genomic organization of SARS-CoV-2

**FIGURE 1:**
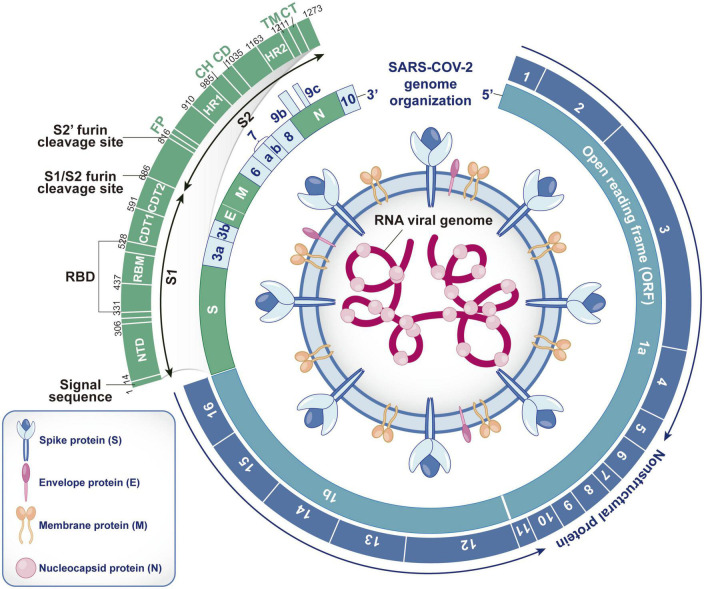
Structure, RNA genome and encoding protein of SARS-CoV-2. SARS-CoV-2 contains spike (S) protein, envelope (E) protein, membrane (M) protein and nucleocapsid (N) protein. The S, E, and M proteins are embedded in the bilayer phospholipid envelope, and the RNA genome is located in the center, wrapped by N protein. The RNA genome of SARS-CoV-2 consists of 15 open reading frames (ORFs), which can encode 16 non-structural proteins (Nsp1-16), 4 structural proteins (S, E, M, and N) and 9 auxiliary proteins (ORF3a, ORF3b, ORF6, ORF7a, ORF7b, ORF8, ORF9b, ORF9c, and ORF10). As the most important surface protein of SARS CoV-2, S protein is mainly composed of S1 and S2 functional subunits and plays a key role in the process of virus invasion and replication. (S1, receptor-binding subunit; S2, membrane fusion subunit; NTD, N-terminal domain; RBM, receptor binding motif; RBD, receptor binding domain; CDT1&CDT2, C-terminal domain; FP, furin peptide; HR1&HR2, heptad repeats; CH, central helix; CD, connector domain; TM, transmembrane domain; CT, cytoplasmic tail).

Severe acute respiratory syndrome coronavirus 2 is a single- and positive-strand RNA virus with an envelope. As a highly pathogenic coronavirus, it belongs to Betacoronavirus (Kadam et al., 2021). The genome sequence of SARS-CoV-2 (approximately 29.9 kb in size) has approximately 80 and 50% homology to those of SARS-CoV and MERS-CoV, respectively (Arya et al., 2021). Two-thirds of the genome contains 15 open reading frames (ORFs), which encode 16 non-structural proteins (Nsp1-16) to form replicase-transcriptase complexes (Rahnavard et al., 2021). Studies have shown that Nsp1 can hinder ribosome binding to host mRNA and inhibit protein synthesis in host cells, Nsp2 can promote virus replication by inhibiting the autophagy defense mechanism of host cells and destroying mitochondrial function, and Nsp3 can cleave the N-terminus of the polyprotein to produce independent functional Nsp1 and Nsp2 and reduce the antiviral ability of host cells by changing the protein balance. Nsp4 uses the plasma membrane of the endoplasmic reticulum to mediate the formation of vesicle structures, Nsp5 participates in the cleavage of other Nsps after synthesis, Nsp6 interacts with Nsp3 and Nsp4 to promote the formation of virus replication organelles with double-membrane vesicle structures, Nsp7 and Nsp8 form hexadecameric complexes and act as primer enzymes allowing Nsp12 to stabilize viral RNA, Nsp10 can activate the 2′-O-methyltransferase activity of Nsp16, and Nsp12 is a catalytic subunit with RNA-dependent RNA polymerase activity that can catalyze viral RNA synthesis and assist in viral RNA replication in combination with Nsp7 and Nsp8. Nsp13 can untangle the entangled viral RNA strand and facilitate subsequent viral RNA replication. Nsp14 has a 3′–5′ exonuclease domain, which can enhance the proofreading ability during viral RNA replication. Nsp15 can remove viral RNA fragments to minimize the antiviral defense response of host cells. Nsp16 is a 2′-O-ribose methyltransferase that methylates the 5′ cap of viral RNA and inhibits its degradation by cells (Yadav et al., 2021; Yang and Rao, 2021; Yoshimoto, 2021; Bai et al., 2022). The remaining third of the genome encodes nine accessory proteins (ORF3a, ORF3b, ORF6, ORF7a, ORF7b, ORF8, ORF9b, ORF9c, and ORF10) and four structural proteins [spike (S) protein, envelope (E) protein, membrane (M) protein and nucleocapsid (N) protein] (Rahnavard et al., 2021). Accessory proteins are not necessary for virus replication (Gao X. et al., 2021), but they regulate innate immunity and promote viral infection. The S protein is the most important surface protein of SARS-CoV-2 and is closely related to its transmission ability. The N protein is abundant in SARS-CoV-2, is a highly immunogenic protein and participates in genome replication and cell signaling pathway regulation (Arya et al., 2021; Figure 1).

The S protein plays an important role in the virus subtype and vaccine response and is responsible for the entry of SARS-CoV-2 into host cells (Seyran et al., 2021). The S protein is mainly composed of the S1 and S2 functional subunits. The S1 subunit is composed of the N-terminal domain (NTD) and receptor-binding domain (RBD), and its function is to recognize receptor proteins of host cells. The S2 subunit mainly consists of a fusion peptide, heptapeptide repeat sequence 1 and central helix, which are responsible for assisting the membrane fusion process between the virus and host cells (Seyran et al., 2021). Therefore, it is very important to understand the receptor recognition mechanism of SARS-CoV-2, which determines its infectivity, host range and pathogenesis.

## 3. SARS-CoV-2 infection mediated by ACE2

After the structural characteristics of the SARS-CoV-2 membrane fusion protein were determined, ACE2 was identified as its main binding receptor. Hoffmann et al. further found that the S protein of SARS-CoV-2 is the key to viral invasion into cells. In contrast to other coronaviruses (CoVs), the S protein of SARS-CoV-2 has a site composed of multiple arginine residues at the junction of the S1 and S2 subunits, which can be recognized and cleaved by Furin (Xia et al., 2020). In fact, the S protein is cut by Furin in the Golgi apparatus of the host during biosynthesis, which is the premise of SARS-CoV-2 infecting host cells (Walls et al., 2020). Usually, mature S protein exists in a metastable prefusion conformation. To bind to the receptor, the RBD in the S1 subunit undergoes a hinged conformational motion between two states of “up” and “down”. When the RBD is in the “up” state, it can bind to the receptor ACE2; in the “down” state, it cannot (Yan et al., 2020). The three-dimensional structure of the S protein is observed by cryo-electron microscopy. Only one RBD is in the “up” conformation, and the other two are in the “down” conformation without binding activity. The binding of one RBD in the “up” conformation with ACE2 leads to the other two RBDs adopting unstable “up” conformations (Wrapp et al., 2020), which further destroys the stability of the prefusion trimer and promotes the transformation of the S protein from a metastable prefusion conformation to a stable fusion conformation.

**FIGURE 2:**
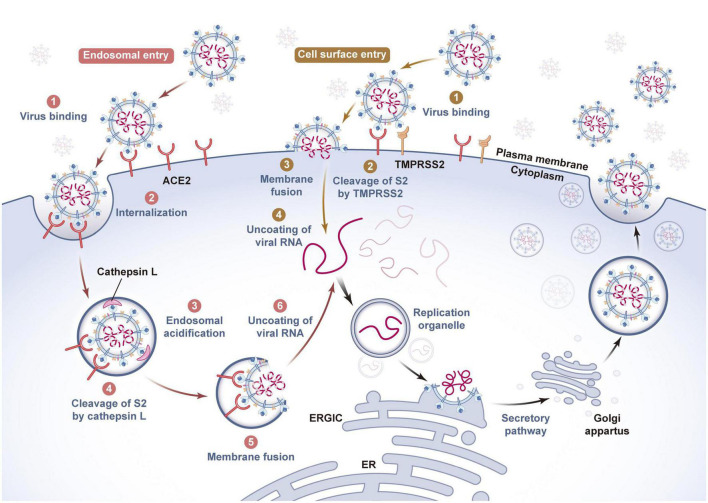
Invasion and release mechanism of SARS-CoV-2 based on ACE2 receptor. The host cells infected by SARS-CoV-2 mainly include virus adsorption, invasion, genetic material release, genome replication and transcription, assembly, budding and other processes. As a key protein for SARS-CoV-2 to recognize host cells, spike protein has a strong binding force with the cell membrane surface receptor ACE2. There are two fusion pathways for SARS-CoV-2 to invade host cells, one is viral envelope-endosome membrane fusion pathway mediated by endocytosis, and the other is viral envelope-cell membrane fusion pathway. When the viral genome is released into the cytoplasm of the host cells, it will induce the endoplasmic reticulum to form a replication organelle with a double membrane structure, complete genome RNA replication and structural protein synthesis, and assemble and generate new viral particles at the endoplasmic reticulum of the host cells, which will be transported by golgi to the host cell membrane and released to the outside of the cell by exocytosis. The whole process is critical to the survival and pathogenicity of SARS-CoV-2, and requires the participation of host cell proteases such as Furin, TMPRSS2 and Cathepsin L. (ACE2, angiotensin-converting enzyme II; TMPRSS2, transmembrane serine protease 2; ER, endoplasmic reticulum; ERGIC, endoplasmic reticulum- golgi intermediate compartment).

Studies have confirmed that SARS-CoV-2 mainly infects host cells through membrane fusion and endosomal fusion. When the RBD successfully recognizes and binds ACE2, it causes the S1 subunit to detach and the S2 subunit to adopt a highly stable postfusion conformation, thus performing its membrane fusion function. If enough cell surface protease transmembrane serine protease 2 (TMPRSS2) can be induced to cleave the S2′ site, FP in the exposed S2 subunit is inserted into the host cell membrane at the same time. Then, heptad repeat 1 (HR1) and HR2 gradually approach, narrowing the distance between the virus outer membrane and host cell membrane. Finally, HR1 and HR2 are induced to form an 6-helix bundle structure in an alternating reverse parallel arrangement so that the virus envelope fuses directly with the cell membrane and releases genetic material. In contrast, if the RBD is not recognized and cleaved by TMPRSS2 on the cell surface after binding with ACE2, the SARS-CoV-2-ACE2 complex enters the cell through the reticulin-mediated endocytosis pathway, and the S2′ site is cleaved by Cathepsin (Cat) B/L in the endosome. Then, the virus envelope fuses with the endosome membrane, and the virus RNA genome is released into the cytoplasm (Frolova et al., 2022; Jackson et al., 2022). In summary, after the S protein binds to the receptor, the effectiveness of proteases such as TMPRRS2, Cat B/L and Furin on target cells largely determines how the virus enters host cells. In the absence of TMPRSS2 expression, Cat B/L is very important for SARS-CoV-2 infection mediated by the S protein. In addition, because SARS-CoV-2 has a unique Furin cleavage site and the proteolytic activation of Cat B/L depends on endosome acidification, while the entry pathway mediated by TMPRSS2 is not affected by pH, SARS-CoV-2 utilizes the TMPRSS2-dependent membrane fusion pathway to infect human cells (Ou et al., 2021; Figure 2).

To sum up, SARS-CoV-2 infection mediated by ACE2 is a multimolecular interaction process, and the complexity of this process means that inhibitors developed for a single target do not necessarily have reliable curative effects in clinical treatment and cannot be further popularized. Therefore, it is necessary to further clarify the role of other receptors and proteases of SARS-CoV-2 in of viral infection and to find more virus-host interaction mechanisms specific to SARS-CoV-2 to clarify why the virus has strong infection and transmission ability. This is not only helpful to fully understand the biological characteristics of the virus but also provides a basis for the treatment of COVID-19 and accelerates research on rapid diagnosis and targeted drugs.

## 4. SARS-CoV-2 infection mediated by other receptors

For the overall concept of SARS-CoV-2 receptors on cell membranes, the prerequisite is whether the relevant receptors can directly interact with S proteins and undergo conformational changes. However, some viruses rely not only on receptors and suitable host cells but also cell surface molecules that may help viruses locate and enter host cells but do not play a decisive role in the infection process and are therefore called attachment factors (coreceptors) (Evans and Liu, 2020). Therefore, virus receptors can be roughly divided into two categories: entry receptors and attachment factors (Mercer et al., 2020). As early as the beginning of the epidemic, researchers found that in the process of SARS-CoV-2 infection, the entry of virus into host cells does not depend on the binding of a single receptor with the S protein but requires the participation of multiple transmembrane proteins in target cells, which also play an important role in the process of virus transmission and can enhance the attachment and invasion ability of SARS-CoV-2 to host cells (Qi et al., 2020). At present, ACE2 is still recognized as the main entry receptor of SARS-CoV-2-infected host cells. However, the expression distribution of ACE2 in human tissues is relatively limited. In virus-positive tissues such as the brain, heart, liver and even lung, ACE2 is expressed in only a small number of cells, and the expression level is low (Scialo et al., 2020), which obviously cannot fully explain the multiorgan tropism of SARS-CoV-2. Therefore, researchers speculate that other receptors may help SARS-CoV-2 enter host cells and have been exploring in this direction. Here, we list a series of important viral receptors and outline their roles in SARS-CoV-2 infection, hoping to further reveal the pathogenesis of SARS-CoV-2 and show the potential value of these protein receptors in the treatment of COVID-19.

### 4.1. Entry receptors

#### 4.1.1. AXL

Although it has been confirmed that ACE2 is the main receptor for SARS-CoV-2 entering human cells, studies have shown that ACE2 is only expressed in the kidney and digestive system, and its overall expression in human lungs and trachea is low. Therefore, there may be other receptors that promote SARS-CoV-2 entry into respiratory system cells. Wang S. et al. (2021) confirmed this hypothesis, and SARS-CoV-2 can invade the respiratory system by using the tyrosine-protein kinase receptor UFO (AXL) protein in host cells. They found that AXL is highly expressed in H1299 and BEAS-2B cells and can directly combine with the NTD of the virus S protein, enter host cells by the reticulin-mediated endocytosis pathway, and colocalize in early endosomes. Moreover, the experimental results also show that there is no cross-inhibition function between ACE2 and AXL. ACE2 knockout has no effect on SARS-CoV-2 infection of H1299 cells, while AXL knockout significantly weakens the viral infection of host cells. It is clear that AXL is a new receptor of SARS-CoV-2 and can independently mediate viral infection of the human respiratory system without relying on ACE2. In addition, in HEK293T cells with both ACE2 and AXL knockout, overexpression of AXL alone can significantly promote viral infection, while the addition of soluble recombinant AXL protein or recombinant NTD protein can inhibit AXL-mediated viral infection, which further confirms that AXL-mediated SARS-CoV-2 infection cannot depend on ACE2. In a word, AXL is a novel entry receptor of SARS-CoV-2 that can significantly promote viral invasion and reproduction and plays an important role in SARS-CoV-2 infection of the human respiratory system. Moreover, AXL can also be used as a potential target for future COVID-19 targeted therapy.

#### 4.1.2. TfR

In a breakthrough in SARS-CoV-2 research, transferrin receptor (TfR) has been proven to be another receptor that affects the entry of SARS-CoV-2 into the host. Researchers found that the expression of TfR in the lung tissue and trachea of SARS-CoV-2-infected monkeys and humanized ACE2 (hACE2) mice increased significantly (Tang et al., 2020). There is a direct interaction between TfR and SARS-CoV-2, and the colocalization signal can be observed in the membrane and cytoplasm of Vero-E6 cells infected by SARS-CoV-2, which indicates that TfR is a membrane receptor of SARS-CoV-2. More importantly, the colocalization signals of TfR, ACE2 and S proteins were also detected on the membrane of infected cells, but only the TfR-S protein complex was observed in the cytoplasm. In other words, TfR can transport SARS-CoV-2 from the cell membrane to the cytoplasm independently of ACE2. Similarly, in Vero E6 and A549 cells with ACE2 knockout, overexpression of TfR can significantly promote viral infection, while knocking down TfR can inhibit viral infection, which once again indicates that TfR can mediate SARS-CoV-2 entry into host cells independently of ACE2. In addition, adding antibodies or polypeptides designed based on the TfR amino acid sequence showed good protective effects *in vitro* and *in vivo*, which could effectively prevent pathological lung injury caused by SARS-CoV-2 infection. These results indicate that TfR is the entry receptor of SARS-CoV-2 infection, and its discovery will further expand our understanding of the SARS-CoV-2 infection mechanism and provide new clues for exploring the therapeutic targets of COVID-19.

#### 4.1.3. ASGR1 and KREMEN1

Systematic analysis of the host cell receptor lineage of SARS-CoV-2 and the search for unknown functional receptors are of great importance for further exploring the complexity of SARS-CoV-2 in tropism and clinical manifestations. Accordingly, Gu et al. (2022) established a genome-wide secretory omics interaction screening system and screened the receptors or ligands of target proteins under physiological conditions. The results turned out that asialoglycoprotein receptor 1 (ASGR1) and kringle containing transmembrane protein 1 (KREMEN1) could interact with multiple domains of the SARS-CoV-2 S protein. ASGR1 can bind to the RBD and NTD, and KREMEN1 can bind to the RBD, NTD and S2 subunits. Further *in vitro* infection experiments confirmed that overexpression of ASGR1 and KREMEN1 can promote SARS-CoV-2 pseudoviral infection in ACE2 knockout cells, indicating that ASGR1 and KREMEN1 can act as entry receptors in ACE2-independent cell lines and directly mediate SARS-CoV-2 infection independent of ACE2. The difference is that they have no effect on SARS-CoV and MERS-CoV infection, and there is no binding site between ASGR1 and the S protein of the virus, which indicates that ASGR1 and KREMEN1 are specific receptors of SARS-CoV-2. In addition, ACE2, ASGR1 and KREMEN1, which can mediate SARS-CoV-2 invasion, are collectively called ASK entry receptors, and different ACE2/ASGR1/KREMEN1 receptor combinations are used to mediate SARS-CoV-2 entry into different types of cells. The results showed that the ASK receptors together constitute the molecular basis of SARS-CoV-2 cell and tissue tropism. Moreover, the correlation between ASK receptor expression and virus susceptibility is obviously stronger than that of any single entry receptor at both the cellular and tissue levels. Moreover, SARS-CoV-2 can invade different types of cells by using different receptors, among which ASGR1 plays a role in liver cell lines, KREMEN1 plays a role in lung cell lines, and ACE2 plays a wide role in both lung and liver cell lines. In addition, the researchers also developed blocking monoclonal antibodies against ASGR1 and KREMEN1. The research findings suggested that these antibodies could significantly inhibit SARS-CoV-2 infection in human lung organs. More importantly, compared with any single targeting antibody, a “cocktail antibody” targeting ASK receptors can significantly inhibit SARS-CoV-2 infection of lung organs. In conclusion, ASGR1 and KREMEN1, as entry receptors of SARS-CoV-2, play an important role in SARS-CoV-2 infection independent of that of ACE2. This study provides important information and clues for explaining and deeply understanding the tropism and pathogenesis of SARS-CoV-2 and provides new targets and treatment strategies for drug research and development against COVID-19.

#### 4.1.4. Integrin

One of the main pathological symptoms of many severe COVID-19 patients is platelet abnormalities, such as thrombocytopenia, microvascular thrombosis, and abnormal coagulation function. Kuhn et al. (2023) conducted in-depth exploration of the reasons. Their research confirmed that Integrin is the most abundant receptor protein on the surface of platelets (does not express ACE2 receptor). Among them, Integrins α5β1 and αvβ3, as the entry receptors of SARS-CoV-2, can directly recognize and bind to the RGD ligand motif (arginine-glycine-aspartic protein sequence) in the RBD domain of the S protein and do not depend on ACE2-mediated viral infection of platelet cells. In addition, the interaction between the S protein and Integrin αvβ3 can trigger the remodeling of Actin in cells and induce the formation of filamentous pseudopodia on the cell surface. These processes further activate platelets, ultimately leading to extensive microthrombosis and poor prognosis. Similarly, the infection of T cells by SARS-CoV-2 and related immune responses are closely related to the prognosis of COVID-19. Severe patients often exhibit symptoms such as T-cell overactivation, apoptosis, and lymphocyte reduction in the clinic. However, there is almost no expression of ACE2 and other known SARS-CoV-2 receptors on T cells, and how SARS-CoV-2 infects T cells and triggers their functional abnormalities has always been a focus of attention in the field of coronavirus research. The work of Huang et al. (2023) further clarified this problem, and their research results showed that lymphocyte Integrin α4β1, α4β7, α5β1, and αLβ2, as newly identified receptors for SARS-CoV-2, can directly recognize the three binding motifs in the S protein RBD and mediate viral entry into T cells. After integrin activation, the binding ability of RBD to these Integrins is significantly enhanced, which can significantly promote SARS-CoV-2 infection of T cells. In addition, the combination of RBD and Integrin further triggered the phosphorylation of Src and Akt proteins in T cells and upregulated the membrane expression level of the activation molecule CD25, as well as the inflammatory factors interleukin-2, interferon-γ and tumor necrosis factor-α at the transcriptional level, in turn inhibiting T-cell proliferation. In a word, SARS-CoV-2, under the direct effect of lymphocyte Integrin, enters T cells to regulate the T-cell immune response and inhibit cell proliferation, which is one of the important reasons for the high-level inflammatory environment and lymphocyte reduction in the body. As an entry receptor for SARS-CoV-2 on T cells, lymphocyte Integrin may be a potential therapeutic target in COVID-19 treatment.

### 4.2. Attachment factors

#### 4.2.1. NRP1

Daly et al. (2020) from Bristol University confirmed that SARS-CoV-2 can bind to a cell surface protein called neuropilin-1 (NRP1). This attachment factor is widely expressed in lung cells and olfactory cells and has the highest expression level in endothelial cells. It was further found that in HEK293T cells with almost no endogenous expression of ACE2 and NRP1, overexpression of NRP1 did not affect the infection degree of SARS-CoV-2 pseudovirus, but coexpression of NRP1, ACE2 and TMPRSS2 significantly promoted viral entry and infection. Similarly, in Caco-2 cells with endogenous expression of ACE2, the overexpression of NRP1 significantly increased the degree of pseudoviral infection. These results suggest that NRP1 can promote the key linkage between the SARS-CoV-2 S protein and ACE2 receptor and the infectivity of SARS-CoV-2 in the presence of the ACE2 entry receptor. In addition, immunoprecipitation and other techniques showed that NRP1 could directly bind to the RRAR amino acid sequence on the S1 subunit of the virus. In contrast, SARS-CoV-2 infection can be significantly inhibited by blocking this interaction with RNA interference technology, monoclonal antibodies and selective inhibitors. Cantuti-Castelvetri et al. (2020) also found that SARS-CoV-2 infected NRP1-expressing cells in the nasal cavity in the pathological analysis of olfactory epithelium obtained from autopsies of deceased COVID-19 patients. Importantly, SARS-CoV-2 infection was also detected in oligodendrocyte transcription factor 2 (OLIG2)-positive cells, which are mainly expressed by olfactory neuron progenitor cells, which can reconstruct axons of the nose and brain when we lose our sense of smell (caused by SARS-CoV-2 infection), so the above reconstruction pathway may be used by SARS-CoV-2 to invade the nervous system. These results provide a new perspective for the treatment of COVID-19, and blocking the binding of SARS-CoV-2 to ACE2 and NRP1 will become a valuable treatment strategy.

Bone marrow macrophages (BMMs) are the main source of osteoclasts, as they can differentiate into osteoclasts by cell fusion. In a recent study (Gao et al., 2022), Liu et al. (2022) found that SARS-CoV-2 can efficiently infect BMMs through viral infection experiments in mice and primary BMMs, leading to bone system damage. Further mechanistic studies showed that the expression of ACE2 in BMMs was very low, while the expression of another receptor, NRP1, was positively correlated with SARS-CoV-2 infection. Moreover, TMPRSS2 was minimally expressed in BMMS, but Cat B/L was highly expressed, with a close relationship to NRP1 expression, suggesting that SARS-CoV-2 probably entered BMMS through the Cat B/L-mediated endosomal pathway. Notably, contrary to previous research reports, SARS-CoV-2 infection of BMMs only depends on the NRP1 receptor, without the participation of ACE2. In addition, SARS-CoV-2 infection of BMMS can significantly inhibit their differentiation into osteoclasts, resulting in abnormal bone resorption and destroyed bone homeostasis. Overall, this study provides the first important evidence for SARS-CoV-2 infection mediated by NRP1 in BMMs for the first time and establishes a potential relationship between osteoclast differentiation disorder and skeletal system metabolism disorder in COVID-19 patients.

#### 4.2.2. CD147

CD147, also known as Basigin or EMMPRIN, belongs to the immunoglobulin superfamily. Early as in the study of SARS-CoV and other viruses (Faghihi, 2020), CD147 was proven to promote viral invasion of host cells, and CD147-antagonistic peptide-9 was shown to exert a significant inhibitory effect on SARS-CoV infection. Subsequently, researchers used CD147 as a candidate target and conducted a series of studies on the spread of SARS-CoV-2. In the research of Wang Z. et al. (2020), it was found that CD147, as an attachment factor, can help SARS-CoV-2 enter host cells through endocytosis. In this study, antiviral detection was carried out *in vitro*. The above research indicated that adding meplazumab (humanized anti-CD147 antibody) could significantly inhibit SARS-CoV-2 infection of host cells and significantly promoted the rehabilitation of infected patients. Afterward, the researchers verified the binding between CD147 and the S protein, and the results of immunoelectron microscopy showed that the two proteins were colocalized in Vero E6 cells infected by SARS-CoV-2. Overall, CD147, as a coreceptor, is a newly identified way for SARS-CoV-2 to infect host cells and provides an important target for the development of specific antiviral drugs. In addition, patients with severe and critical COVID-19 are often likely to have pulmonary fibrosis. Another study by the team showed that CD147 was involved in SARS-CoV-2-induced pulmonary fibrosis in addition to mediating SARS-CoV-2 into the host and initiating the COVID-19 cytokine storm (Wu et al., 2022). CD147 has been identified as the key regulatory factor of SARS-CoV-2- and bleomycin-induced fibroblast activation. Knockdown of CD147 can directly inhibit TGF-β-stimulated lung fibroblast activation and thus reduce susceptibility to bleomycin-induced pulmonary fibrosis. In contrast, the application of meplazumab can significantly inhibit the accumulation of activated fibroblasts and the production of extracellular matrix proteins, thus alleviating the further development of pulmonary fibrosis caused by SARS-CoV-2. In a word, the above studies indicate that CD147 can promote SARS-CoV-2-induced progressive pulmonary fibrosis and provide a theoretical and experimental basis for formulating treatment strategies for fibrosis symptoms.

#### 4.2.3. KIM1

Autopsy and virological studies found many virus inclusion bodies and particles in the kidneys of COVID-19 patients (Wysocki et al., 2020), which indicated that SARS-CoV-2 had renal tissue tropism, and the kidney was another organ susceptible to SARS-CoV-2 in addition to the lung. However, ACE2 inhibitors did not significantly improve the prognosis of COVID-19 patients, and the expression level of ACE2 in the kidney decreased significantly after viral infection, which suggested that there may be other receptors of SARS-CoV-2 in the kidney. In view of the above conjecture, Yang et al. (2021) proposed that kidney injection molecule-1 (KIM1) is an attachment factor of SARS-CoV-2, which can mediate the invasion of the virus into the kidney. Studies have confirmed that KIM1, which is significantly upregulated during kidney injury, can bind with the RBD of SARS-CoV-2, enabling the virus to better attach to the cell membrane and enter cells through reticulin-mediated endocytosis. Moreover, the viral RBD can bind to KIM1 and ACE2 through different binding pockets, suggesting that these two receptors may exert a synergistic effect to mediate SARS-CoV-2 infection. In addition, based on the interaction sequence between KIM1 and SARS-CoV-2, the team designed two polypeptides to block viral infection, among which AP2, a polypeptide consisting of 14 amino acids, can significantly reduce the aggregation of SARS-CoV-2 on the cell surface. Importantly, the team also proposed the theory of “malignant circulation” for SARS-CoV-2 invasion of the kidney; that is, in the early stage of SARS-CoV-2 invasion of the kidney, it mainly binds ACE2 (the expression level is higher than that of KIM1 in the physiological state), and after virus-induced acute kidney injury, the expression level of KIM1 is upregulated, thus promoting secondary viral infection mediated by KIM1 and ACE2, aggravating kidney injury and further upregulating KIM1. In conclusion, the expression level of KIM1 is upregulated only after kidney injury, which indicates that it is relevant and specific to renal function. At the same time, KIM1 was identified as a new target for SARS-CoV-2 to invade cells, providing an important theoretical basis for the development of polypeptide drugs, small molecule drugs and antibody drugs based on this target to treat COVID-19.

#### 4.2.4. SR-B1

Scavenger receptor class B type I (SR-B1), as the core of the lipid delivery system, has been widely recognized in hepatocytes, ovarian cells and testicular interstitial cells. It can mediate the selective absorption of cholesterol esters and play an important role in the metabolism of high-density lipoprotein (HDL) and the “reverse transport” of cholesterol (Powers and Sahoo, 2022). A research result from several cooperative teams confirmed that the S1 subunit of SARS-CoV-2 has specific affinity for HDL, and SR-B1 acts as the cell surface receptor of HDL, making HDL a “bridge” between the virus S protein and SR-B1 receptor (Wei et al., 2020). Therefore, in host cells expressing ACE2, the increase in HDL can significantly promote the adhesion and invasion of SARS-CoV-2 to the cell surface. In contrast, HDL-enhanced SARS-CoV-2 infection can be directly inhibited when cultured cells are treated with a monoclonal antibody (blocking the HDL binding site on the S1 subunit) or SR-B1 antagonist *in vitro*. In addition, to further explore the relationship between SR-B1 and SARS-CoV-2 infectivity, SR-B1 overexpression and knockdown were shown to increase and decrease virus RNA levels in host cells, respectively. In addition, immunohistochemical analysis showed that SR-B1 and ACE2 could be coexpressed on the cell surface of many susceptible tissues (such as lung, retina, kidney, small intestine, colon, etc.), suggesting that SR-B1 may be a coreceptor of ACE2 and enhance the infectivity of SARS-CoV-2 in various susceptible organs and tissues. In general, this study reveals for the first time that SR-B1 is an attachment factor of SARS-CoV-2, which not only clarifies the relationship between SARS-CoV-2 and lipid metabolism but also helps to improve people’s understanding of the pathogenesis of SARS-CoV-2 and provides a theoretical basis for screening and developing new antiviral drugs.

#### 4.2.5. HSPG

Heparan sulfate (HS) is a kind of linear polysaccharide with electronegativity. It connects with the core protein *in vivo* to form a heparan sulfate proteoglycan (HSPG) structure, which exists in the cell membrane and extracellular matrix of mammalian tissue. Infection usually begins with the virus attaching to the glycan on the cell surface. Therefore, to further understand the binding of spike protein and sugar molecules, Tang et al. (2020) used glycan microarray and surface plasmon resonance techniques to test the binding differences of S proteins, subunits and domains of SARS-CoV-2 (Hao et al., 2021), SARS-CoV and MERS-CoV to different HS. The research results show that the S protein of these three human coronaviruses can bind to HS, and the position and degree of sulfation of HS are the key factors affecting its binding. The more glucosamine 6-O-sulfate groups and the higher the sulfation degree, the more easily viral protein binds to HS. The main reason for this is that the expression levels of sulfate transferases vary in different tissues or cells of the same tissue at different stages, resulting in diversity in the structure and function of HS linked by the same core protein. HSPG exhibits different sulfation patterns in different tissues, developmental stages, and pathological states, and this binding specificity may contribute to the tropism of SARS-CoV-2 for human cells. In contrast, the chain length and monosaccharide composition of HS have little effect on binding. Overall, HSPG binding is the molecular basis mediating viral attachment to host cells. Similarly, in the research of Clausen et al. (2020), HSPG was confirmed to exist on the surface of lung cells, and SARS-CoV-2 can adhere to host cells with low affinity in advance by combining with HSPG, thus increasing the virus concentration on the surface of the cell membrane. Moreover, the combination of the two can also induce structural changes in the S protein, forming an “open” conformation, further enhancing the interaction between RBD and ACE2 and initiating the viral infection program. Importantly, SARS-CoV-2 entering lung cells must bind HSPG and ACE2 located on the cell surface at the same time. In addition, removing HSPG with an enzyme or using heparin (competitive binding S protein) can prevent SARS-CoV-2 from binding to HSPG on the cell surface, hinder the adsorption of virus to host cells, and thus inhibit viral infection. To sum up, HSPG is a necessary coreceptor in the process of SARS-CoV-2 infection that can help the virus gradually form aggregates on the cell surface. Moreover, HSPG can enhance the openness of RBD and promote its interaction with receptors, thereby triggering the fusion pathway between virus and cell membrane. Therefore, it is very important to a deep understanding of the pathogenic mechanism of SARS-CoV-2 in human cells for the prevention and treatment of COVID-19.

#### 4.2.6. MYH9

Severe acute respiratory syndrome coronavirus 2 is highly contagious because the virus can infect host cells in many ways. To further explore the specific mechanism by which SARS-CoV-2 infects human lung tissue cells, Chen et al. (2021) jointly identified the receptor protein myosin heavy chain 9 (MYH9) interacting with the virus S protein and confirmed that the binding of the two proteins was realized by the direct binding of the C-terminal domain of MYH9 (named PRA) to the NTD of the S2 subunit and S1 subunit of SARS-CoV-2. In addition, researchers have found that knocking out the MYH9 gene in the wild-type human lung cancer cell lines A549 and Calu-3 by CRISPR/Cas9 technology can significantly inhibit SARS-CoV-2 infection. In contrast, overexpression of MYH9 or PRA enhanced viral infection in wild-type A549 and H1299 cells. Notably, endosome or myosin inhibitors can effectively block SARS-CoV-2 from entering lung cells, but TMPRSS2 and Cat B/L inhibitors are ineffective, which indicates that MYH9 promotes the endocytosis of SARS-CoV-2 and bypasses the TMPRSS2 and Cat B/L pathways. Equally importantly, overexpression of MYH9 did not enhance SARS-CoV-2 pseudoviral infection in ACE2 knockout A549 cells but only enhanced viral infection in wild-type A549 cells. Therefore, the presence of ACE2 is necessary for the entry of SARS-CoV-2 mediated by MYH9. MYH9 can act as a coreceptor for SARS-CoV-2 to enter the host and enhance viral infection by promoting the endocytosis of SARS-CoV-2 dependent on ACE2, but this process does not require the participation of TMPRSS2 and the CatB/L pathway. Overall, MYH9 plays a key role in SARS-CoV-2 infection in cells with low expression of ACE2 (lung tissue cells), and it may be another important potential target for future clinical intervention strategies.

#### 4.2.7. C-type lectins and TTYH2

Excessive lung immune inflammation caused by SARS-CoV-2 entering the human body is considered the main driving factor aggravating COVID-19. Therefore, clarifying the immune pathogenesis caused by SARS-CoV-2 is the key to adopting correct intervention strategies for disease treatment. As previously reported (Liao et al., 2020), researchers detected the existence of viral RNA in immune cells (especially myeloid cells) isolated from alveolar lavage fluid of COVID-19 patients. However, the expression level of the ACE2 receptor molecule in immune cells is very low, which suggests that there may be other receptor molecules mediating the interaction between SARS-CoV-2 and immune cells. In response to this question, Xie et al. cooperated to provide a new explanation (Lu et al., 2021). Researchers have identified six myeloid cell membrane proteins that bind to the virus S protein, which can be used as attachment factors of SARS-CoV-2. Among them, c-type lectins (DC-SIGN, L-SIGN, LSECtin, ASGR1 and CLEC10A) mainly bind to the non-RBD (NTD or CTD) domain of the S protein, while tweety family member 2 (TTYH2), similar to ACE2, mainly binds to the RBD domain of the S protein. However, further virus verification experiments found that the interaction between SARS-CoV-2 and these surface receptors could not affect viral infection and replication but could cause myeloid cells to produce large amounts of proinflammatory cytokines (IL1B, IL8, CXCL10, CCL2, etc.) and upregulate the expression of inflammation-related genes (EGR1, THBD, C4A, and SOCS3). Moreover, RNA sequencing analysis of single cells in alveolar lavage fluid of COVID-19 patients showed that these inflammatory factors were not only closely related to the severity of symptoms but also positively related to the expression of receptors on the surface of myeloid cells. In addition, the researchers designed a bispecific nanoantibody (aiming at two blocking modes of myeloid cell receptor and ACE2 receptor), which can not only effectively prevent SARS-CoV-2 infection mediated by ACE2 but also block the excessive inflammatory reaction caused by SARS-CoV-2 through myeloid cell receptor. In total, this study reported for the first time that the interaction between SARS-CoV-2 and immune cell surface receptors can lead to excessive inflammatory reactions and proposed using bispecific nanoantibodies to block the viral infection pathway and immune overactivation at the same time as a potential treatment strategy for severe COVID-19 patients.

#### 4.2.8. GRP78

Glucose regulated protein 78 (GRP78) is a molecular chaperone protein that usually exists in the endoplasmic reticulum of cells. However, new research suggests that GRP78 is also localized on the cell surface, serving as an attachment factor for SARS-CoV-2. On the target cell surface, it can promote the viral endocytosis by directly interacting with the S protein and endogenous ACE2 receptor (Shin et al., 2022). Notably, the soluble form of GRP78 can exist in the systemic circulation, especially in COVID-19 patients, and its protein expression level significantly increases with increasing severity of SARS-CoV-2 infection. Moreover, soluble GRP78 can form complexes with viral particles in the body circulation or plasma membrane, thus enhancing the stability of the virus and further promoting the adhesion and invasion of SARS-CoV-2 to the host cell surface. Moreover, GRP78 plays a key role as on exogenous viral protein. It can act as the host virus chaperone of SARS-CoV-2 proteins (such as S, E, N, NSPs, and ORFs) and participate in processes such as the folding, assembly and degradation of viral proteins through its own chaperone function (Nassar et al., 2021). In addition, the continuous mutation of the virus and its own adaptability have always been key issues that urgently need to be addressed in anti-SARS-CoV-2 treatment. To further explore whether targeting GRP78 can treat COVID-19, researchers tested a new small molecule drug HA15 (GRP78 inhibitor) on infected lung cells (Ha et al., 2022). The results indicate that this drug can specifically bind to GRP78 and inhibit its activity, effectively reducing the number and size of SARS-CoV-2 plaques produced in infected cells. The researchers also carried out similar studies in animal models infected with SARS-CoV-2. The results showed that HA15 significantly reduced the viral load in the lungs, and the safe dose had no harmful effect on normal cells. Surprisingly, the original purpose of this drug development is to combat cancer. This targeted inhibitor will build a bridge between cancer treatment and COVID-19 treatment. It may be possible to treat different diseases together.

#### 4.2.9. DPP4

Dipeptidyl peptidase-4 (DPP4) is a serine protease that exists on the cell surface in the form of dimer. It is widely distributed in human tissues and functions as a multifunctional protein. DPP4 is expressed in the respiratory tract, kidneys, liver, small intestine, and central nervous system. In addition, it is widely expressed on the surface of activated immune cells, including CD4 + T cells, CD8 + T cells, B cells, NK cells, dendritic cells and macrophages. It can regulate the production of cytokines, chemokines and peptide hormones, thus participating in various immune or inflammatory reactions. A bioinformatics study based on the protein crystal structure revealed that DPP4 can serve as a cell surface binding target for the virus S protein RBD (Li et al., 2020) and enhance SARS-CoV-2 infection of otherwise non-susceptible cells by promoting ACE2 receptor-dependent endocytosis, and mutation of E484 and its adjacent residues is an important factor in this binding ability. Notably, camostat mesylate (serine protease inhibitor) has been proven to be effective in inhibiting SARS-CoV-2 infection. Since DPP4 is a serine protease, DPP4 inhibitors may also be developed as therapeutic drugs targeting COVID-19. In addition, another study related to DPP4 inhibitors found that diabetes patients are more likely to be infected with SARS-CoV-2 and develop severe cases of COVID-19; moreover, SARS-CoV-2 can change the expression of DPP4 in diabetes patients through interaction with insulin, leptin and interleukin-6 (IL-6) and trigger uncontrolled glucose metabolism disorder and inflammation, thereby increasing the mortality of COVID-19 (Rakhmat et al., 2021). Due to the important role of DPP4 in glucose metabolism, the application of DPP4 inhibitors can help reduce the severity of the disease through this pathway, preventing lung inflammation and reducing lung injury. In general, the potential use of DPP4 as a binding target (coreceptor) for SARS-CoV-2 may provide new insights into the pathogenesis of the virus and help develop monitoring and treatment strategies to address the challenges of COVID-19.

#### 4.2.10. Other coreceptors

In addition to the attachment factors mentioned above, the following coreceptors have been confirmed to play key roles in SARS-CoV-2 infection. The susceptibility of cells to viruses may be closely related to interaction with these receptor proteins, which can ensure the triggering of SARS-CoV-2 attachment and invasion of the host at appropriate locations. For example, T-cell immunoglobulin and mucin domain 1 (TIM-1) and TIM-4, as important phosphatidylserine (PS) receptors in the TIM and TAM families, can directly interact with PS on the outer leaflet of SARS-CoV-2, enhancing viral infection by promoting ACE2-dependent SARS-CoV-2 endocytosis fusion (Bohan et al., 2021). A disintegrin and metalloprotease 17 (ADAM17), as one of the coreceptors for SARS-CoV-2 entering the host, is mainly triggered by the virus S protein to cleave ACE2, further leading to extracellular detachment of ACE2 and enhancing host protease activity, promoting virus cytoplasmic fusion (Jocher et al., 2022). Similarly, the attachment factor B^0^AT1 (SLC6A19) can enhance the stability of ACE2 and assemble it into a high-quality and stable heterodimer structure, and the ACE2-B^0^AT1 complex can simultaneously bind two S proteins, significantly promoting SARS-CoV-2 recognition and infection of host cells (Yan et al., 2020). In addition, the S protein can directly bind to the pattern recognition receptor toll-like receptor 4 (TLR4) to enhance viral attachment on the surface of host cells, increase the virus concentration on the membrane surface, activate the downstream signal of TLR4 and upregulate the related inflammatory factors IL-1B and IL-6, thus inducing the body’s antibacterial-like natural immune response (Zhao et al., 2021). In contrast, there are also some receptors on the host cell that inhibit viral entry. Previous research results have shown that interferon-induced transmembrane protein 3 (IFITM3) is a protein that can prevent SARS-CoV-2 from passing through the cell membrane. Its main mechanism is to prevent the fusion of the viral envelope and cell plasma membrane by regulating the fluidity of the host cell membrane, thus preventing viral invasion (Xu et al., 2022). Lymphocyte antigen-6E (LY6E) is a glycosyl phosphatidylinositol-anchored cell surface receptor that also plays a role in body defense. It inhibits SARS-CoV-2-induced cell infection by interfering with S protein-mediated membrane fusion and cytoskeleton rearrangement (Rajah et al., 2022). The ezrin protein receptor is encoded by the EZR gene, which can inhibit viral infection of the host by reducing the expression of key receptors (ACE2 and TLR) related to SARS-CoV-2. Ezrin peptide has also been proven to be particularly effective in inhibiting viral pneumonia and is a key way to prevent and treat severe COVID-19 (Gadanec et al., 2021). These reports provide new insights into the interaction between SARS-CoV-2 and the host. Further research on this aspect can help us better understand the host tropism and pathogenicity of SARS-CoV-2 and provide a theoretical basis and potential targets for the prevention and treatment of COVID-19.

## 5. SARS-CoV-2 is mutating

### 5.1. Alpha

The Alpha variant, numbered B.1.1.7, was first identified in the UK in September 2020 and began spreading rapidly in mid-December (Schuit et al., 2021). In addition to the mutation D614G in the S protein, in the RBD of the S protein of the Alpha mutant, asparagine at position 501 was replaced by tyrosine (N501Y), which increased the affinity between the RBD and ACE2. This resulted in the Alpha variant being able to infect cells with lower ACE2 levels than nasal and bronchial epithelial cells. Although the loss of amino acid 144 on the NTD of the S protein did not cause significant structural rearrangement, it led to drug resistance to most anti-NTD monoclonal antibodies (Domingo and de Benito, 2021; Mohammad et al., 2021). Therefore, compared with the original strain, the main feature of the Alpha variant strain is its ability to combat the immune system, which enhances not only transmissibility but also virulence and can lead to breakthrough of the existing natural immunity of humans and to secondary infection (Peters et al., 2021).

### 5.2. Beta

The Beta variant, numbered B.1.351, was first discovered in South Africa in May 2020 and soon became the most widespread variant strain in South Africa (Bhattarai et al., 2021). In addition to the N501Y mutation in the S protein, lysine at amino acid position 417 in the RBD is replaced by asparagine (K417N), and glutamate at amino acid position 484 is replaced by lysine (E484K). Among them, the N501Y mutation enhances receptor recognition, and the additional K417N and E484K mutations facilitate immune evasion, which promotes resistance to neutralization by therapeutic monoclonal antibodies against the RBD. In addition, the replacement of leucine at position 18 with phenylalanine (L18F) and the loss of amino acids at positions 242, 244, and 243 causes rearrangement of the NTD epitope, which significantly reduces the antiviral efficacy of monoclonal antibodies against the NTD (Sutton et al., 2022; Yadav et al., 2022). Therefore, the main features of the Beta variant strain are vaccination-induced immunity escape, elevated transmissibility, and evasion of immune cell recognition, thus reducing the protection afforded by COVID-19 vaccines (Bhattarai et al., 2021; Liang et al., 2022).

### 5.3. Gamma

The Gamma variant, numbered P.1, was first discovered in Brazil in November 2020, and it is the most important SARS-CoV-2 strain in South America (Loconsole et al., 2021). Compared with the sequence of the Beta mutant, in the S protein RBD of the Gamma mutant, lysine at position 417 is replaced with threonine (K417T), while the E484K and N501Y mutations are retained. In addition, there is an L18F mutation in the NTD, and threonine at position 20 is replaced by asparagine (T20N), proline at position 26 is replaced by serine (P26S), etc., (Raman et al., 2021; Yépez et al., 2022). Therefore, for the ACE2 receptor, the Gamma variant has a binding affinity similar to that of the Beta variant, and mutation at these sites significantly reduces the neutralizing efficacy of monoclonal antibodies against the RBD and NTD. The main characteristic of the Gamma variant is that it can evade immunity and transmit more readily. Its transmissibility is twice that of the original strain, and some people can still undergo reinfection after recovering from infection by the Gamma strain (Brinkkemper et al., 2021).

### 5.4. Delta

The Delta variant, numbered B.1.617.2, was first discovered in India in October 2020, and it spread globally, causing major outbreaks in India, Britain and other countries (Bhattacharya et al., 2022). The Delta mutant also carried the D614G mutation. In addition, in the S protein RBD, leucine at position 452 is replaced by arginine (L452R), threonine at position 478 is replaced by lysine (T478K), threonine at position 19 is replaced by arginine (T19R), glycine at position 142 is replaced by aspartic acid (G142D), the amino acids at positions 156 and 157 are missing, etc. Among them, the L452R mutation is closely related to the increased infectivity of the delta variant and reduction of neutralization by convalescent plasma and specific monoclonal antibodies (Wilhelm et al., 2021; Dhawan et al., 2022). Therefore, the main characteristics of the Delta variant are fast transmission speed, high viral load and strong adaptability to the body. Statistical analysis shows that Delta has the strongest infection ability among all variant strains at present, and its infection rate is twice that of the Alpha strain and 1,260 times that of the original strain (Yi et al., 2022). Even more worrisome is that the condition of patients infected with the Delta variant progresses rapidly, and the average time to the severe stage is only 5 days (Zhan et al., 2022).

### 5.5. Omicron

The Omicron variant, numbered B.1.1.529, was first discovered in South Africa in November 2021, becoming the fifth VOC, and it has gradually replaced Delta and become prevalent worldwide (Rana et al., 2022). The Omicron variant contains more than 50 mutations, including 37 mutation sites on the S protein alone, representing the most frequent mutation in the S protein among variants at present (Bazargan et al., 2022). Importantly, the Omicron variant incorporates the other four key mutation sites of the S protein of other VOCs, and there are 15 amino acid mutation sites in the RBD region, including K417N, T478K, and N501Y, among which E484A and Q493R are unique to Omicron. Among these mutations, the K417N, N501Y, Q493R, and Y505H mutations can enhance the binding ability of the virus to ACE2 and thus increase its infectivity. Mutations such as S477N, T478K, and E484A may enhance the immune evasion ability of viruses, thus avoiding the surveillance of the immune system (Khandia et al., 2022; Tian et al., 2022). Furin, a highly specific endonuclease, can enhance the pathogenicity of viruses. There are three mutations (H655Y, N679K, and P681H) near the furin cleavage site of the Omicron variant, which may enhance the cleavage of the S protein and its fusion with host cells and further promote the replication and transmission of the virus (Bansal and Kumar, 2022). In addition, the R203K/G204R mutation in the N protein is closely related to an increase in viral RNA expression and viral load, which may enhance viral virulence (Singhal, 2022). Therefore, the main features of the Omicron variant strain are its strong transmissibility and immune evasion ability. Omicron is called a supervariant strain, and the number of cases due to Omicron is rising much faster than that of previous SARS-CoV-2 outbreaks. Fortunately, it has less virulence and causes low mortality, and some infected people have no symptoms (Trunfio et al., 2022).

In the early stages of the Omicron epidemic, three subtypes emerged, namely, BA.1-3, with BA.2 having the strongest epidemic intensity (Tiecco et al., 2022). Compared with the previous subtypes, Omicron subtype BA.4 and BA.5 have stronger cell fusion ability in alveolar epithelial cells, so with the accelerated spread of BA.4/5 in many countries, the world set off the seventh wave of the epidemic surge (Tallei et al., 2022). XBB is a recombinant strain of Omicron BA.2.10.1 and BA.2.75, with stronger transmission and immune escape ability than the early prevalent Omicron variant (Vogel, 2023). It is currently the dominant epidemic strain in multiple countries worldwide. XBB.1.5 was first found in the United States in October 2022 and has been marked as the most infectious SARS-CoV-2 subtype variant thus far, with at least 26 subbranches (XBB.1.5.1∼XBB.1.5.10) (Channabasappa et al., 2023). According to the data released by the US Centers for Disease Control and Prevention (CDC) website, during the week of March 12-18, 2023, cases of XBB.1.5 accounted for 90.2% of the total number of cases of COVID-19, and the virus strain developed from a low-frequency epidemic to an absolute advantage epidemic strain in less than 3 months (Center for Disease Control and Prevention [CDC], 2023). XBB.1.9 was first detected in Indonesia in September 2022 and currently has three sub branches (XBB.1.9.1 to 1.9.3) (Velavan et al., 2023). Notably, although there has been an increase in cases of XBB.1.9 worldwide recently and its transmission potential is higher than that of XBB.1.5, individuals who have been infected with XBB.1.5 will not be infected with XBB.1.9 again in the short term, which greatly suppresses its spread (World Health Organization [WHO], 2022b). Recently, a new strain FU.1 (XBB.1.16.1.1) with stronger infectivity has emerged in multiple regions worldwide. Experts have warned that the danger of FU.1 is very high, with 50% higher infectivity than the current epidemic strain, and it has only just begun to spread globally (Herald Sun, 2023). In short, although the COVID-19 epidemic no longer constitutes a “public health emergency of international concern,” it is still a serious infectious disease, and we need to consider it a serious threat to human health.

## 6. Effectiveness of the COVID-19 vaccines against the Omicron variants

**FIGURE 3:**
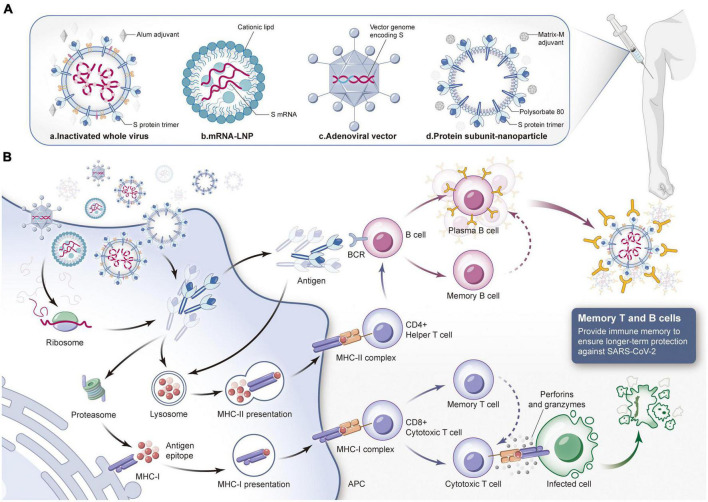
Composition of different types of COVID-19 vaccines and the mechanism of protective immune response induced by them. **(A)** Different types of COVID-19 vaccines. a. Inactivated virus vaccine: Use physical and chemical methods to inactivate SARS-CoV-2 and add specific adjuvants. The inactivated virus injected into the body has no pathogenicity but only retains immunogenicity. b. mRNA vaccine: Lipid nanoparticles are used as delivery carriers to introduce mRNA expressing spike (S) protein into the body to produce neutralization reaction. c. Virus vector vaccine: Integrate S protein gene into adenovirus genome to construct recombinant adenovirus vaccine, and then inject it into muscle. d. Protein subunit vaccine: The S protein or its subunit fragment prepared *in vitro* is mixed with a specific adjuvant and injected into the human body to induce the immune response of the body. Then stimulate the immune system to produce specific immune response against SARS-CoV-2. **(B)** The human immune system will produce immune response against the invading vaccine components. When COVID-19 vaccine is injected into the body, it can be internalized by antigen presenting cells, and S protein or its subunit fragments can be transmitted or synthesized in the cytoplasm. If the target antigen is decomposed into small fragments by the proteasome complex, MHC-I molecules can present the antigen fragments to the cell surface to facilitate the recognition of CD8^+^cytotoxic T cells. Activated cytotoxic T cells kill infected cells by secreting lymphokines such as perforin and granzyme. If the target antigen secreted is reabsorbed by cells, it will be degraded by lysosomes and presented to CD4^+^helper T cells through MHC-II molecules on the cell surface. B cells will be activated by the stimulation of antigen, proliferate and differentiate into plasma cells, secrete specific antibodies, and the antibodies can combine with SARS-CoV-2 to make them lose their infectivity. At the same time, they will guide macrophages to phagocytosis and eliminate pathogens. In addition, both memory T cells and memory B cells have the ability to recognize specific antigens. If they encounter the same target antigen invasion again in the future, they can be quickly activated to kill the invading pathogens. (LNP, lipid nanoparticle; APC, antigen-presenting cell; MHC, major histocompatibility complex; BCR, B cell receptor).

At present, COVID-19 continues to spread in many places worldwide, and vaccination is one of the most effective strategies for pandemic prevention. According to the data released by the WHO on March 30th, 2023, there are 183 vaccines in clinical trials and 199 vaccines in preclinical trials (World Health Organization [WHO], 2023). These vaccines mainly include subunit vaccines (32%), inactivated vaccines (12%) and attenuated live vaccines (1%), as well as emerging mRNA vaccines (24%), viral vector vaccines (14%), DNA vaccines (9%), and virus-like particle vaccines (4%) (Li G. et al., 2022), among which 11 vaccines are included in the Global Emergency Use List (EUL) (Table 2). However, SARS-CoV-2 variants appear frequently, and whether the existing COVID-19 vaccines can effectively slow the spread of the current pandemic variants has become a global issue of general concern to the public. Here, we focus on the latest research progress of these 11 vaccines from the perspective of the technical route, working mechanisms, advantages and disadvantages of the vaccines and neutralization efficacy against Omicron pandemic strains, hoping to further improve the rationale and accuracy of COVID-19 prevention and control measures (Figure 3).

**TABLE 2:** Comparison of 11 COVID-19 vaccines included in the WHO emergency use list.

Types of vaccines	Representative vaccines	Fundamental	Advantages	Disadvantages
Inactivated virus vaccine	BBIBP-CorV/ Coronavac/ Covaxin	The virus is cultured *in vitro* and inactivated by physicochemical methods. The inactivated virus only retains immunogenicity and causes specific immune response as antigen	Mature technology, easy preparation and large-scale production. The inactivated virus does not replicate in the host and is not contagious	The immunogenicity is insufficient and adjuvant needs to be added. The immune effect period is short and multiple injections are needed. Weak ability to cope with virus mutation
mRNA vaccine	BNT162b2/ mRNA-1273	The mRNA expressing S protein is introduced into the body through a specific delivery system, and the S protein is expressed in the body and induced to produce specific immune response	Short research and development cycle, low production cost, and timely response to the outbreak of mutant strains. No virus component, no risk of infection	Poor stability and high requirements for storage and transportation conditions. There is virulence risk. Large-scale production process needs to be optimized
Viral vector vaccine	Vaxzevria/ Covishield/ Ad26.COV2.S/ Ad5-nCoV	The S protein gene is inserted into the modified adenovirus genome which is harmless to the body, and then inject it into the human body to stimulate the body to produce specific immune response	No adjuvant is needed, and the adverse reactions are small. The number of vaccinations is less, which can induce immune response faster and is suitable for large-scale population vaccination	The body may have pre-existing immunity to adenovirus vector, which will reduce the immune effect of vaccine
Subunit vaccine	Covovax/ Nuvaxovid	The S protein or its protein fragment is prepared directly *in vitro* by genetic engineering recombination method, and then inject it into vivo to induce specific immune response	High safety and good stability. Easy storage and transportation	The immunogenicity is weak, and additional adjuvants are needed to enhance it

### 6.1. Inactivated virus vaccines

Inactivated vaccine technology is recognized as a mature classic technology worldwide. In its preparation, the virus strain should be first isolated, amplified and cultured in susceptible cells and then inactivated, purified and adsorbed by an adjuvant so that the original virus strain loses pathogenicity and retains antigenicity. Subsequently, helper T cells can be activated by antigen-presenting cells, and then B cells can be induced to produce humoral immunity (Wang Z. et al., 2020). Although inactivated virus particles can no longer infect, they contain the complete structural proteins of the virus, which can simultaneously induce the production of polyclonal antibodies against various SARS-CoV-2 antigens (such as the S protein and N protein). However, due to the complexity of virus culture and inactivation procedures, the development and production of inactivated vaccines requires more time (Khoshnood et al., 2022). Moreover, the immune protection duration is relatively short, and two or even multiple invasive injections are required (Khoshnood et al., 2022).

According to the up-to-date information from the WHO, three inactivated vaccines have been granted emergency use authorization, including BBIBP-CorV produced by the Beijing Institute of Biological Products Co., Ltd. and CoronaVac produced by Beijing Kexing Biological Products Co., Ltd. Covaxin was developed by Bharat Biotech (World Health Organization [WHO], 2022a). BBIBP-CorV is the first COVID-19 vaccine listed by the WHO for emergency use in China. In the early stage of the widespread pandemic of the Omicron variant, Professor Zhang Wenhong and Professor Wang Pengfei of Fudan University jointly evaluated the immune evasion ability of the Omicron variant after two doses of BBIBP-CorV and the third booster vaccination. Studies showed that the antibody titer level of the subjects vaccinated with two inactivated vaccines against the Omicron strain decreased by at least 5.3-fold compared with that against the original strain. However, after the third booster injection, the antibody titer increased significantly, and the antibody-positive rate reached at least 75% (Ai et al., 2022). CoronaVac is the most widely administered COVID-19 vaccine in the world. The latest study comprehensively analyzed the immune response induced by the third booster dose of the CoronaVac injection. The strong immune memory could be quickly recalled by the third injection, and the neutralizing activity of serum against the original strain, Delta variant strain and Omicron variant strain increased by 4.08-fold, 5.0-fold, and 3.6-fold, respectively. Subsequently, Chen R. et al. (2022) also dynamically monitored specific cellular immunity against SARS-CoV-2. It was found that the RBD protein-specific B cells and memory B cells for the original strain induced by the third injection could also cross-recognize the RBD of Delta and Omicron variants. Regarding Covaxin, the WHO recently issued a statement that it would suspend the procurement and supply of Covaxin due to manufacturing practice defects found during inspection and suggested that countries receiving this vaccine “take appropriate actions” (Thiagarajan, 2022).

### 6.2. mRNA vaccines

As a powerful tool against the COVID-19 pandemic, mRNA vaccines have been widely studied. These vaccines can introduce mRNA expressing antigen targets into the body through a specific delivery system, and then the proteins are translated and expressed, whereupon they effectively stimulate the body to produce specific immune responses (a dual humoral immunity and T-cell immunity mechanism) to achieve efficient immune protection (Fang et al., 2022). The mRNA vaccine uses the gene sequence of SARS-CoV-2, containing viral component and carrying no risk of infection. In addition, mRNA vaccines have short research and development cycles, simple production processes and easy mass production, which can improve the global vaccination rate. More importantly, for the constantly mutating SARS-CoV-2, once a new variant sequence is determined, researchers can develop a new vaccine to deal with the mutant strain in the shortest time by modifying the mRNA sequence of the target. However, mRNA molecules have a short half-life and poor stability and are extremely sensitive to high temperature, which makes vaccine storage and transportation difficult (Jin et al., 2021; Szabó et al., 2022).

Since the emergence of COVID-19, the medical prospects of mRNA vaccines have been fully proven. At present, mRNA vaccines included in the EUL by the WHO include BNT162b2, which was jointly developed by Pfizer/BioNTech, and mRNA-1273, which was produced by Moderna (World Health Organization [WHO], 2022a). Both vaccines were developed with the design strategy of wrapping mRNA encoding the SARS-CoV-2 S spike protein with lipid nanoparticles (LNPs) (Noori et al., 2022), and vaccinees are afforded good protection. A study led by Professor Lu Ligong found that among people who have been vaccinated with two doses of an inactivated virus vaccine, those who choose BNT162b2 for the booster injection will effectively produce more neutralizing antibodies against Omicron (Li G. et al., 2022). Similarly, Muecksch et al.’s (2022) research shows that compared with people who received only 2 shots of an mRNA vaccine, those who received a third shot of mRNA-1273 exhibit enhanced ability of the body to produce broad-spectrum neutralizing antibodies, and more than 50% can neutralize Omicron variants. In addition, as reported Moderna Company, the first mRNA vaccine, mRNA-1273.214 specifically targeting Omicron will be launched soon, covering 32 mutations of the S protein and exhibiting an excellent neutralizing effect on Omicron (BA.4/5). Compared with that before mRNA-1273 booster vaccination, the neutralizing antibody level increased by 8-fold, and the geometric mean titer (GMT) of antibodies increased from 432 to 3,070 1 month after mRNA-1273.214 booster vaccination (Chalkias et al., 2022).

### 6.3. Viral vector vaccines

Viral vector vaccines use improved and safe viruses (such as adenovirus, HIV, etc.) as vectors to deliver genes encoding viral protective antigens to host cells (Deng S. et al., 2022), thus activating the human body to produce specific neutralizing antibodies and develop immune protection. There are two kinds of viral vector vaccines. One is non-replicative; the virus retains its complete structure and infectivity, but its self-replication function is lost. It needs the help of specific transformed cells or helper viruses to stimulate the human body to produce effective immunity. The other is the replication type, which can also replicate and produce more vector viruses to complete a new round of infection, express more specific antigens, and then trigger a stronger immune response (Jacob-Dolan and Barouch, 2022). The viral vector vaccines approved for SARS-CoV-2 protection are all recombinant vaccines based on adenovirus as the delivery vector and S protein as the target. Its advantage lies in the high transgenic efficiency of the adenovirus vector, which can transduce different types of human tissues and cells, and the vaccine can be inoculated with only one injection, with an average of 14 days to achieve immune protection. Therefore, it is time-saving, convenient, efficient and suitable for rapid large-scale vaccination of populations (Chen L. et al., 2022). However, because adenovirus vectors are generally susceptible, most humans have antibodies to neutralize adenovirus, which leads to the immune system attacking viral vectors, thus reducing the effectiveness of the vaccines (McCann et al., 2022).

Globally, four kinds of adenovirus vaccines have been authorized by the WHO for emergency use. These vaccines are Vaxzevria and Covishield, which were jointly developed by Oxford University/AstraZeneca, Ad26.COV2.S developed by Johnson & Johnson and Ad5-nCoV produced by Consino Biological AG (World Health Organization [WHO], 2022a). Research results have proven that although the preventive effect of the vaccine decreased compared with that against the original strain, individuals initially immunized with 2 doses of Vaxzevria still retained neutralizing activity against Omicron. After the third-dose booster injection, the titer of neutralizing antibody against Omicron increased significantly (Hastert et al., 2022). During the fourth COVID-19 wave in South Africa triggered by the Omicron strain, Gray Glenda’s team conducted mass spectrometry analysis on serum samples from critically ill patients who were vaccinated with 2 doses of Ad26.COV2. The research findings suggested that Ad26.COV2.S booster injection could effectively prevent hospitalization with severe COVID-19, and the effective rate was 55% 14 days after inoculation, 74% 14–28 days after inoculation, and 72% 1–2 months after inoculation. The curative effect of critically ill COVID-19 patients admitted to the ICU is also very significant. The effective rate was 82% 1-2 months after vaccination (Gray et al., 2022). The researchers randomly divided 904 subjects who had been vaccinated with 2 doses of inactivated vaccine 6 months prior into 3 groups, namely, the 1-dose Ad5-nCoV heterologous boosted group, 1-dose ZF2001 heterologous boosted group and 1-dose CoronaVac homologous boosted group. The data showed that 14 days after booster vaccination, the neutralizing antibody GMT against the Omicron strain of the Ad5-nCoV group was 261 and that of the ZF2001 group and the CoronaVac group was 86 and 54, respectively. In addition, the positive rate of IFN-γ production in the Ad5-nCoV heterologous boosted group was 68.8%, and the cellular immune response intensity was significantly better than that in the other two groups (Sapkota et al., 2022). Therefore, Ad5-nCoV was selected as the booster injection for heterologous boosting, which is the best choice for protective immune effects at present.

### 6.4. Subunit vaccines

A protein subunit vaccine is a new vaccine that can induce immune protection based on the recombination of the protective antigen gene of the virus with a plasmid or viral vector and introducing it into a recipient cell expression system, inducing the production of specific antigen proteins and purifying them (Golob et al., 2021). Subunit vaccines usually employ the S protein or RBD protein of SARS-CoV-2 as candidate vaccine antigens to eliminate epitopes with poor immune effects. This approach can not only improve the utilization efficiency of antigen epitopes but also avoid the production of unrelated antibodies and reduce the side effects of vaccines (Mekonnen et al., 2022). On the other hand, because such a vaccine needs only the recombination and expression of effective antigen fragments *in vitro* without any live virus, vaccine production can be conducted in an ordinary biosafety laboratory, which significantly reduces the production cost, and the storage and transportation of the vaccine are more convenient, enabling large-scale production capacity (Díaz-Dinamarca et al., 2022; Heidary et al., 2022). However, because this kind of vaccine includes only a part of the virus structure, its immunogenicity is relatively low, so it is necessary to add appropriate adjuvants to enhance the immune effect of the vaccine.

With the ongoing development of vaccines against COVID-19, subunit vaccines have become the largest category of vaccine research and development. NVX-CoV2373 (Covovax and Nuvaxvid) is a protein subunit vaccine jointly developed by Novavax/Serum Institute of India and was listed by the WHO for emergency use on December 17 and 21, 2021, respectively (World Health Organization [WHO], 2022a). Early in the Omicron outbreak, Novavax released raw data on the cross-reaction of NVX-CoV2373 for Omicron. The results showed that NVX-CoV2373 produced an extensive cross-reactive immune response to Omicron and other SARS-CoV-2 variants. Compared with that after the initial immunization with two doses of NVX-CoV2373, the body produced a stronger immune response after the third-dose booster injection, and the anti-S protein IgG antibody and ACE2-binding inhibitory antibody levels increased by 9.3 times and 19.9 times, respectively (Tarke et al., 2022). In addition, recent data have shown that NVX-CoV2373 can produce an effective immune response to many Omicron subtypes, including BA.5, which has the strongest immune evasion ability (Zeng et al., 2022). Overall, NVX-CoV2373 plays an important role in fighting against the new SARS-CoV-2 variants.

### 6.5. Other types of vaccines

In addition to the abovementioned COVID-19 vaccines, other types of vaccines have been developed, tested and approved for use. For example, the working mechanism of DNA vaccines belonging to the third-generation technical approach is to introduce DNA fragments encoding antigen proteins into host cells, which then continuously express natural antigens *in vivo* to induce the human body to produce a sustained and effective immune response (Wang X. et al., 2022). Compared with mRNA vaccines, DNA vaccines have higher stability and are easier to produce and store, but they may also integrate DNA into the host chromosome to cause mutations and induce autoimmune diseases (Hadj Hassine, 2022). At present, only ZyCoV-D developed by Zydus Cadila has been authorized for emergency use and approved for vaccination for people over 12 years old. It is not only the first DNA COVID-19 vaccine officially approved for marketing but also the first DNA vaccine applied to humans in the world (Khobragade et al., 2022). In fact, more than a dozen DNA COVID-19 vaccines are undergoing clinical trials, and INO-4800, which was jointly developed by Inovio and Aidi Weixin, has made the fastest progress and attracted the most attention (Kraynyak et al., 2022). Virus-like particles (VLP) vaccines is a kind of nano particles formed by self-assembly of one or more structural proteins of a virus. It has similar spatial structure and composition with natural virus particles, and can effectively induce immune protective response in human body. Because they do not contain viral genetic material, VLP vaccines are not infectious and have relatively high safety. However, their immunogenicity is insufficient. Similar to subunit vaccines, they need adjuvants and multiple immunizations to enhance the immune effect, and their preparation is complex and difficult (Tariq et al., 2021). CoviFenz (VLPs based on plants), a COVID-19 vaccine jointly developed by GSK/Medicago, has been officially approved by the Canadian Ministry of Health for marketing. This vaccine is the first approved plant-derived COVID-19 vaccine (Hemmati et al., 2022).

Attenuated vaccines consist of pathogen variants with weakened or no virulence obtained by attenuating pathogens by specific methods before inoculating them into the human body to induce an immune response (Okamura and Ebina, 2021). In contrast to inactivated virus vaccines, an attenuated vaccine retains the replication ability and immunogenicity of the original strain, so its immune effect is stronger, and its action time is longer. However, there may be potential pathogenic risks in immunocompromised people vaccinated with attenuated vaccines. More importantly, because the attenuation and screening of pathogens in the early stage takes a large amount of time, it is very difficult to complete the research and development of products in a short time, which indicates that attenuated vaccines will not be the first choice for managing outbreaks (Chen, 2022). To date, only two candidate vaccines, COVI-VAC and MV-014-212, have been approved for clinical trials (Wang S. et al., 2021; Tioni et al., 2022). Moreover, some COVID-19 vaccines (synthetic peptide vaccines and circular RNA vaccines) are in an accelerated process of research and development (Qu et al., 2022), with the hope that they can produce remarkable protective effects in the clinic.

## 7. Establish a mode to cope with the COVID-19 epidemic

To date, the COVID-19 epidemic has gone from the original virus strain to fifth generation VOCs. Obvious changes have taken place in the genomics, biology and epidemiology of Omicron. The extremely strong transmission and immune evasion ability is accompanied by a decline in the pathogenicity of the virus itself. Combined with the population immune barrier established by previous infection and vaccination, compared with the previous variant strains, the related epidemic shows the characteristics of strong transmission and a low severity rate and mortality rate (Trunfio et al., 2022), which opens a new stage of the COVID-19 epidemic. At the same time, faced with a series of problems, such as Omicron’s influence on epidemic prevention policy, livelihoods and social and economic operations, human cognition and response to the COVID-19 epidemic will also enter a new stage. How can humans coexist with viruses at the lowest cost? How can epidemic prevention strategies be optimized to account for epidemic control, livelihood and the economy? These are common problems faced by all countries in the world. Accordingly, we believe that the ultimate control mode of COVID-19 will be the application of COVID-19 vaccines and COVID-19-specific drugs for prevention and treatment to jointly enhance the ability of human beings to cope with the COVID-19 epidemic. In addition, strengthening the monitoring of mutant strains by means of science and technology and adopting appropriate and effective public health protection strategies are also important links to control the spread of the epidemic. In short, we must be soberly aware that relaxing the prevention and control of the epidemic does not mean letting go of the epidemic; we coexist with SARS-CoV-2 but not allow it to spread freely. Because Omicron is still evolving, no one knows the future epidemic trend and the impact of dominant variants on human beings and society.

### 7.1. Effective promotion of vaccination and innovation

New variants of SARS-CoV-2 frequently appear all over the world, and the Omicron variant shows stronger transmissibility and immune evasion ability than previous strains, which directly leads to a certain degree of decline in the protective efficacy of the early developed vaccines (Zhou et al., 2022). Fortunately, even so, Omicron variants are still within the protection scope of these vaccines, and the protection rate of the vaccines is still higher than the established standard of the WHO (a protection rate of vaccines over 50%) (Fernandes et al., 2022). Many studies have proven that vaccination can reduce the transmission risk of the Omicron variant in the population and effectively reduce the incidence of severe illness and mortality after infection (Fernandes et al., 2022; Guo et al., 2022). Therefore, actively promoting vaccination among social groups and establishing an effective immune barrier are currently the most important means to control the COVID-19 pandemic. However, we must also admit that the current vaccines cannot completely prevent Omicron infection, and we still need to further innovate and develop specific vaccines, expecting the emergence of new vaccines based on Omicron variants that can provide stronger and more lasting protective efficacy.

### 7.2. Promotion of heterologous booster immunization

In the context of the more “cunning” Omicron variant, vaccination against COVID-19 remains one of the most effective pandemic prevention measures. Studies have shown that the level of neutralizing antibodies in people who have completed basic immunization will obviously decrease over time. To better prevent infection caused by the Omicron variant globally, timely booster immunization can rapidly improve the neutralizing antibody level and immune protection ability of recipients, thus producing a better protective effect on the body (Meng et al., 2022). At present, according to whether the technical routes of the booster vaccine and basic immunization vaccine are consistent, booster immunization can be divided into two methods, namely, homologous booster immunization and heterologous booster immunization. Among them, the preventive effect of the latter is more prominent (Deng J. et al., 2022). A clinical study by Al Kaabi showed that people who had received two doses of BBIBP-CorV inactivated vaccine could produce a strong immune response against the Omicron variant after receiving one dose of NVSI-06-07 protein subunit vaccine by heterologous boosting, and their antibody level was 19.5 times that before boosting. Compared with that of subjects who received homologous boosting with the BBIBP-CorV inactivated vaccine, the neutralizing antibody GMT increased by 7.7 times (Al Kaabi et al., 2022). In addition, ARCoVaX is a new COVID-19 mRNA vaccine jointly developed by Aibo Biology, Academy of Military Medical Sciences and Watson Biological. The results of clinical trials showed that when ARCoVaX booster injection was sequentially inoculated after basic immunization with two inactivated vaccines, it induced a strong neutralizing antibody reaction to Delta and Omicron variants, and the neutralizing reaction remained at a high level until 90 days after inoculation. In addition, compared with homologous vaccination with an inactivated vaccine, heterologous vaccination with ARCoVaX can induce a stronger humoral immune response, which can effectively prevent virus escape and severe illness (Liu et al., 2022). On the whole, clinical research on heterologous booster immunization provides a new idea for vaccination strategies for COVID-19 and provides a more accurate basis for strengthening the optimization of immunization strategies.

At present, many countries and regions worldwide have adopted or recommended heterologous immunization as a booster vaccination strategy for COVID-19 vaccines, especially adenovirus vector vaccines, which can effectively strengthen the protection provided by other vaccines and reduce the incidence of severe diseases (Lv et al., 2022). There are two main advantages of heterologous booster immunization: first, different vaccines can complement each other and increase the neutralizing antibody level of recipients; second, everyone’s constitution is different, which may result in side effects for a certain type of vaccine. Vaccination via different technical routes can circumvent this situation (Nguyen et al., 2022). All in all, heterologous booster immunization is expected to improve the intensity, breadth and persistence of the immune response through the combination of vaccines with different mechanisms, provide more comprehensive and powerful immune protection for the body, and further reduce the risk of breakthrough infection caused by the Omicron strain.

### 7.3. Accelerating the research and development of small molecule antiviral drugs

Antiviral drugs are the first choice to treat SARS-CoV-2 infection. Only by completely eliminating SARS-CoV-2 can COVID-19 be cured. Macromolecular antibody drugs can neutralize viruses directly, but they are easily degraded in the digestive tract, so they cannot be taken orally. Most of them are injected and mainly used for inpatients, and there are some problems, such as high research and development costs and difficulty dealing with virus variation (Huang et al., 2022). In contrast, the development of small molecule chemical drugs is of great importance in the environment of repeated global epidemics. The preparation process of small molecule chemical drugs is simple and mature, which can quickly realize mass production to meet the surge in the number of infected people caused by the Omicron outbreak (Sargsyan et al., 2023). Moreover, such drugs are stable, and most of them are taken orally, which not only greatly improves the compliance of patients but also facilitates administration to the vast numbers of outpatient and home patients, reducing the strain on medical resources and ensuring timely treatment of critically ill patients (Hemmati et al., 2022). The whole process of viral infection can be divided into adsorption, invasion, shelling, replication, assembly and release (Biskupek et al., 2022), so interfering with viral invasion and replication processes is an effective direction for antiviral drug action. At present, oral small molecule antiviral drugs used clinically can be divided into two categories according to their targets: one category is ACE2 inhibitors and membrane fusion inhibitors that target the S protein to hinder viral invasion, while the other is RNA polymerase inhibitors and 3C-like protease (3CLpro) inhibitors that target RNA polymerase and 3CLpro to prevent virus replication (Kralj et al., 2021). From the perspective of drug marketing speed, it is difficult to develop ACE2 inhibitors and membrane fusion inhibitors. More importantly, the mutation of SARS-CoV-2 mainly occurs on the S protein, while RNA polymerase and 3CLpro are highly conserved in SARS-CoV-2 and thus undergo less mutation. Therefore, inhibitors of these two enzymes will have lower off-target effects and will remain effective for all variant strains (Chen R. et al., 2022), making them an important focus of future research and development of anti-SARS-CoV-2 drugs.

At present, dozens of small molecule antiviral drugs for treating COVID-19 are being intensively developed worldwide, almost all of which are RNA polymerase and 3CLpro inhibitors. Some of these drugs have made remarkable progress in research and have obtained marketing licenses or emergency use authorization in many countries. For example, Molnupiravir represents a new generation of RNA polymerase inhibitors developed by Merck. As the world’s first oral small molecule drug, it is mainly used for the treatment of severe and mild and moderate adult patients with high hospitalization risk. It was first launched in the UK in November 2021 (Flisiak et al., 2022). One study included 775 adults with mild to moderate COVID-19 as subjects, including patients with basic diseases such as obesity, diabetes or heart disease with higher risk. Oral administration of Molnupiravir (400 mg) for 5 days significantly reduced the hospitalization rate and mortality. The hospitalization rate of the treatment group was 7.3% (no deaths), while that of the control group was 14.1% (8 deaths) (Czarnecka et al., 2022). Similarly, Paxlovid, developed by Pfizer, is one of the most effective oral drugs at present, and was approved by the FDA to be marketed in the United States in December 2021 for patients with severe risk and non-hospitalized mild to moderate COVID-19 pneumonia. Paxlovid is a combination package that consists of two drugs: nematvir inhibits the 3CLpro of SARS-CoV-2, and ritonavir inhibits the Cytochrome P450 3A4 (CYP3A4) enzyme of the human body, preventing nematvir from being metabolized by the human body and maintaining its blood concentration for a long time (Marzolini et al., 2022). The results of clinical trials showed that when Paxlovid was taken within 3 days of symptoms, the hospitalization rate was only 0.8%, and there were no deaths. In the placebo group, the hospitalization rate was 7%, and the mortality rate reached 1.8% (Kueh, 2022). In total, it is very important to develop broad-spectrum, convenient and low-cost specific drugs for the effective prevention and control of the COVID-19 epidemic. With the development and marketing of oral small-molecule antiviral drugs and the comprehensive vaccination of vaccines, mankind will eventually win the war without gunpowder, and the impact of the COVID-19 epidemic on world production and life will eventually be eliminated.

### 7.4. Strengthening the monitoring of mutant strains

Recently, the China CDC reported the opinion that the Omicron variant will not be the last variant (Chinese Center for Disease Control and Prevention, 2022). Therefore, the monitoring of new variants and the possible impacts of transmission, pathogenicity and immune evasion are the focus of current pandemic prevention and control. Real-time fluorescence quantitative PCR nucleic acid detection has high specificity and sensitivity and has become the “gold standard” for diagnosing SARS-CoV-2 infection (Wang H. et al., 2022). However, because SARS-CoV-2 is an RNA virus, variation in the target sequence develops easily, and too low a viral content in the sample to be detected due to weakening of the virus shedding ability and even some inappropriate operations (including sample collection, transportation, storage and processing, etc.) may lead to negative results in qRT-PCR detection (Rai et al., 2021). In fact, there have been patients worldwide who have been tested more than ten times before having a positive result (MacKenzie et al., 2022). Therefore, no one can guarantee that there will be no mutant strain in the future that can avoid the existing nucleic acid detection, and such a variant may be hidden in a remote part of the world.

High-throughput next-generation sequencing (NGS) is a powerful tool to analyze the genetic relationship between viral evolution and disease, track the epidemic situation, develop new therapies and develop vaccines. This technology can obtain the genome sequence information of unknown viruses for the first time, making it possible not only to effectively monitor the mutation of SARS-CoV-2 but also to identify, trace and analyze the virus epidemiology (de Mello Malta et al., 2021), which is of great significance for cutting off transmission route and tracking the transmission situation. In addition, the successful acquisition of the complete sequence of the mutant strain provides the basis for the subsequent establishment of a rapid detection method. For patients with negative nucleic acid detection and highly suspect clinical phenotypes, NGS technology can also be used for further confirmation (Quer et al., 2022). On the other hand, although NGS has obvious advantages of high throughput, high sensitivity and high accuracy, its detection cost is high, the detection process is cumbersome, and professionals are required to analyze sequencing results, which is not suitable for rapid and mass screening and diagnosis, so it is difficult to be widely used (Quer et al., 2022). In summary, SARS-CoV-2 mutations are still uncertain. All countries should enhance their ability to monitor and sequence mutant strains as much as possible and share complete genome sequence data with international public platforms to help each other cope with the evolving COVID-19 epidemic.

### 7.5. Improve self-immunity and adhere to public health protection measures

“It takes a good blacksmith to make steel” (a metaphor indicating that you need your own strength to do anything) is a familiar traditional Chinese saying. People of all ages are generally susceptible to SARS-CoV-2 because it is a novel pathogen, especially elderly individuals with underlying diseases (diabetes, cardiovascular and cerebrovascular diseases, malignant tumors, etc.) that readily cause complications and critical illness after infection and account for the vast majority of deaths (Gao Y. et al., 2021; Laza et al., 2022). This suggests that there may be a close relationship between immunity and symptoms or manifestations of SARS CoV-2 infection and whether it will develop into severe disease. Therefore, we should scientifically strengthen our physical fitness and enhance our own immunity to resist the invasion of viruses into the our body.

Regarding the current situation and trend of global pandemic development, it can be assumed that SARS-CoV-2 variants will persist. From the perspective of viral transmission, viral replication and mutation complement each other. Effectively controlling the number of infected people in society can directly reduce the chances of viral replication and mutation (Chekol Abebe et al., 2022). Therefore, compliance with public health protection measures is key to controlling the spread of the pandemic, especially in areas where SARS-CoV-2 is spreading intensely. In other words, even after vaccination, we cannot relax our vigilance, and we still need to take preventive measures such as mask wearing, frequent ventilation, frequent disinfection, safe social distancing and reducing unnecessary gatherings.

## 8. Conclusion

Although the transmission capacity of SARS-CoV-2 has not abated, and at present, the transmission coefficient (Rt) of the new Omicron variant XBB.1.5 has exceeded 1.6, making it spread rapidly in the United States and other countries (Vogel, 2023), it is encouraging that the pathogenicity of the virus is gradually decreasing. In particular, the current mainstream Omicron mutants account for more than 95% of patients with mild or even asymptomatic infections (Rajpal et al., 2022). For SARS-CoV-2 to survive in the long term, variants will continuously reduce their virulence as they search for new hosts. During the “self-evolution” process, although some “radicals” (sudden increases in virulence) are inevitable, the vast majority of viruses will gradually extend the host incubation period and weaken pathogenicity through mutations to better adapt to survival. From this perspective, SARS-CoV-2 is “intelligent.”

To sum up, this article comprehensively reviews the genome characteristics of SARS-CoV-2, the pathogenesis of multiple membrane receptors including the classical route of ACE2-mediated viral infection, viral mutation, vaccine development, and the “new model” for COVID-19 epidemic. However, because the virus has the characteristics of mutation and immune escape, potential new developments in pathogenesis, membrane receptor types, and potential vaccines and treatment targets may not have been discovered in a timely manner. In addition, some traditional antiviral drugs, including azvudine, have been clinically proven to be effective in blocking the replication of SARS-CoV-2, which means that some drugs used for other diseases may also play an effective antiviral role in COVID-19 infection. These are the limitations of the content of this article. Therefore, we need to analyze and explore the mutation laws and immune escape mechanisms of SARS-CoV-2 from the perspective of viral mutation, which will lay an important genetic foundation for the research and development of specific antiviral drugs and vaccines. In addition, actively integrating traditional antiviral drug and accelerating clinical trials of drug safety will play an important role in quickly finding more mature COVID-19-specific drugs.

In 2023, the COVID-19 pandemic in most countries and regions in the world, including China, is showing a tendency to stabilize, which is the result of the joint efforts of medical staff and people worldwide. However, SARS-CoV-2 has not completely disappeared, so timely monitoring of mutant strains will become particularly important. Notably, even if you are infected with SARS-CoV-2, you do not have to be afraid if you respond rationally. The most important thing is that the public should refrain from discrimination against positively infected people, eliminate prejudice as much as possible, and not let positively infected people bear the consequences of “social death.”

## Author contributions

ZZ, XG, and XW were the major contributors to the writing and revision of the manuscript. YC, YXZ, ZD, and LH collected the related references and participated in discussions. TW, JY, and RZ made substantial contributions to the conception or design of the work. KZ, ZS, and YHZ approved the final version of the manuscript. All authors contributed to the article and approved the submitted version.
